# Metal Ions and Chemical Modification Reagents Inhibit the Enzymatic Activity of Lecithin-Dependent Hemolysin from *Vibrio parahaemolyticus*

**DOI:** 10.3390/toxins14090609

**Published:** 2022-09-01

**Authors:** Francisco Javier Vazquez-Armenta, Uriel Felipe Valdez-Olmos, Aldo Alejandro Arvizu-Flores, Jesus Fernando Ayala-Zavala, Adrian Ochoa-Leyva, Alonso Alexis Lopez-Zavala

**Affiliations:** 1 Departamento de Ciencias Químico Biológicas, Universidad de Sonora, Encinas y Rosales s/n Col. Centro, Hermosillo 83000, Mexico; 2 Coordinación de Tecnología de Alimentos de Origen Vegetal, Centro de Investigación en Alimentación y Desarrollo, A.C. Carretera Gustavo Enrique Astiazarán Rosas, No. 46 Col. La Victoria, Hermosillo 83304, Mexico; 3 Departamento de Microbiología Molecular, Instituto de Biotecnología (IBt), Universidad Nacional Autónoma de Mexico (UNAM), Cuernavaca 62210, Mexico

**Keywords:** *V. parahaemolyticus*, LDH, metal ions, chemical agent inhibition

## Abstract

Lecithin-dependent thermolabile hemolysin (LDH) is a virulence factor excreted by *Vibrio parahaemolyticus*, a marine bacterium that causes important losses in shrimp farming. In this study, the function of LDH was investigated through its inhibition by metal ions (Mg^2+^, Ca^2+^, Mn^2+^, Co^2+^, Ni^2+^ and Cu^2+^) and chemical modification reagents: β-mercaptoethanol (βME), phenylmethylsulfonyl fluoride (PMSF) and diethyl pyrocarbonate (DEPC). LDH was expressed in the *Escherichia coli* strain BL-21, purified under denaturing conditions, and the enzymatic activity was evaluated. Cu^2+^, Ni^2+^, Co^2+^ and Ca^2+^ at 1 mmol/L inhibited the LDH esterase activity by 20–95%, while Mg^2+^ and Mn^2+^ slightly increased its activity. Additionally, PMSF and DEPC at 1 mmol/L inhibited the enzymatic activity by 40% and 80%, respectively. Dose-response analysis showed that DEPC was the best-evaluated inhibitor (IC_50_ = 0.082 mmol/L), followed by Cu^2+^ > Co^2+^ > Ni^2+^ and PMSF (IC_50_ = 0.146–1.5 mmol/L). Multiple sequence alignment of LDH of *V. parahaemolyticus* against other Vibrio species showed that LDH has well-conserved GDSL and SGNH motifs, characteristic of the hydrolase/esterase superfamily. Additionally, the homology model showed that the conserved catalytic triad His-Ser-Asp was in the LDH active site. Our results showed that the enzymatic activity of LDH from *V. parahaemolyticus* was modulated by metal ions and chemical modification, which could be related to the interaction with catalytic amino acid residues such as Ser153 and/or His 393.

## 1. Introduction

Shrimp farming is one of the most important economic-productive activities in aquaculture. *Litopenaeus vannamei* (white shrimp) is the main farmed specie due to its commercial value and good breeding capacity [1,2]. However, emerging diseases caused by several pathogens, such as bacteria, viruses and parasites, cause high mortalities and economic losses [3]. Among the bacterial-related diseases, Vibriosis is related to Acute Hepatopancreatic Necrosis Disease, previously known as Early Mortality Syndrome, which is one of the most concerning diseases due to the high mortality rate (up to 90%) [4].

*Vibrio parahaemolyticus* is a halophilic bacterium distributed in tropical coastal waters around the world and is the causative agent of AHPND and other Vibrio-related diseases [5]. During infection, *V. parahaemolyticus* uses various virulence factors, including adhesins, toxins and secretion systems [6]. The thermostable direct hemolysin (TDH) and TDH-related hemolysin (TRH) (encoded by *tdh* and *trh* genes, respectively) are considered the main virulence factors closely related to human pathogenicity [7]. However, some isolated clinical strains that do not contain *tdh* and/or *trh* genes remain pathogenic, indicating the expression of other virulence factors [8]. An additional toxin, known as thermolabile hemolysin or lecithin-dependent hemolysin (LDH) encoded by the *tlh* gene, has been gaining attention since the expression of *tlh* was strongly upregulated under conditions simulating the human–host intestinal environment [9]. The *tlh* gene is also used as a molecular marker for *V. parahaemolyticus* pathogenic shrimp strains, and among other hemolysins, it is highly expressed during infection in shrimp farming [10]. Additionally, LDH from *Vibrio alginolyticus* (≈90% identity to *Vp* LDH) was a toxic protein causing 100% mortality in zebrafish [11].

This enzyme is broadly distributed among *Vibrionaceae* species and is a molecular marker for clinical and environmental isolates [12]. The *tlh* gene encodes for 418 amino acid residues with a molecular mass of ≈48 kDa; the N-terminal signal peptide is post-transductionally removed, producing a matured protein of ≈40 kDa [13]. LDH possesses hemolytic properties as phospholipase A_2_ activity, hydrolyzing glycerophospholipids that release lysophospholipids that cause cell lysis [14]. Multiple sequence alignment analysis showed that LDH has well-conserved SGNH domain and GDSL motif characteristics of the hydrolase/esterase superfamily [15]. SGNH hydrolase domain contains the catalytic triad Ser153, Asp390, and His393, which is also found in members of the serine-protease family [16]. Ser153 is activated by His393 deprotonating the hydroxyl group and acts as the nucleophile during catalysis, while Asp390 stabilizes the tetrahedral intermediate [16]. Since these residues are critical for the LDH enzymatic activity and consequent hemolytic activity, they could be considered potential targets for chemical modification or metal binding residues acting as inhibitors. Therefore, these actions could diminish the *V. parahaemolyticus* virulence mediated by hemolytic toxins such as LDH. 

Divalent cations could act as enzyme inhibitors since they can bind and form metal complexes with proteins [17]. Structural analysis of metalloproteins reveals that metals (Co^2+^, Ni^2+^, Cu^2+^ and Zn^2+^) bind to enzymes in coordination with sulfur, oxygen, or nitrogen as donor atoms of side chains resulting in a wide variety of binding geometries [18]. Histidine is the most common metal-binding residue through Nε atom of the imidazole ring, followed by cysteine and glutamic and aspartic acids through carboxyl oxygen [16]. Migliorini et al. [19] reported the interactions of metal ions with model peptides. Inhibition of *V. parahaemolyticus* hemolysin has been focused on pore-forming toxins such as TDH and TRH [20,21], but those related to LDH inhibition are scarce. Jia et al. [11] found that the LDH from *V. alginolyticus* was inhibited by divalent cations, such as Zn^2+^, Cu^2+^, Ni^2+^ and others, but no inhibitory effect of monovalent (Na^+^ and K^+^) ions was observed. In addition, chemical modification is an important approach for activating or inhibiting enzyme function. Some chemical reagents can interact with amino acids to stabilize the 3D protein structure as β-mercaptoethanol (βME) acts as a disulfide bond-reducing agent. Additionally, chemical modification of active site amino acids improves or reduces catalytic properties as phenylmethylsulfonyl fluoride (PMSF) and diethylpyrocarbonate (DEPC), which can interact with hydroxyl groups of serine and the imidazole ring of histidine residue, respectively [11,22,23]. Therefore, this work aimed to evaluate the inhibitory effect of metallic ions and chemical modification reagents in the enzymatic activity of recombinant LDH from *V. parahaemolyticus.*

## 2. Results

### 2.1. Effect of Metal Ions and Chemical Reagents on the Enzymatic Activity of LDH

**Figure 1:**
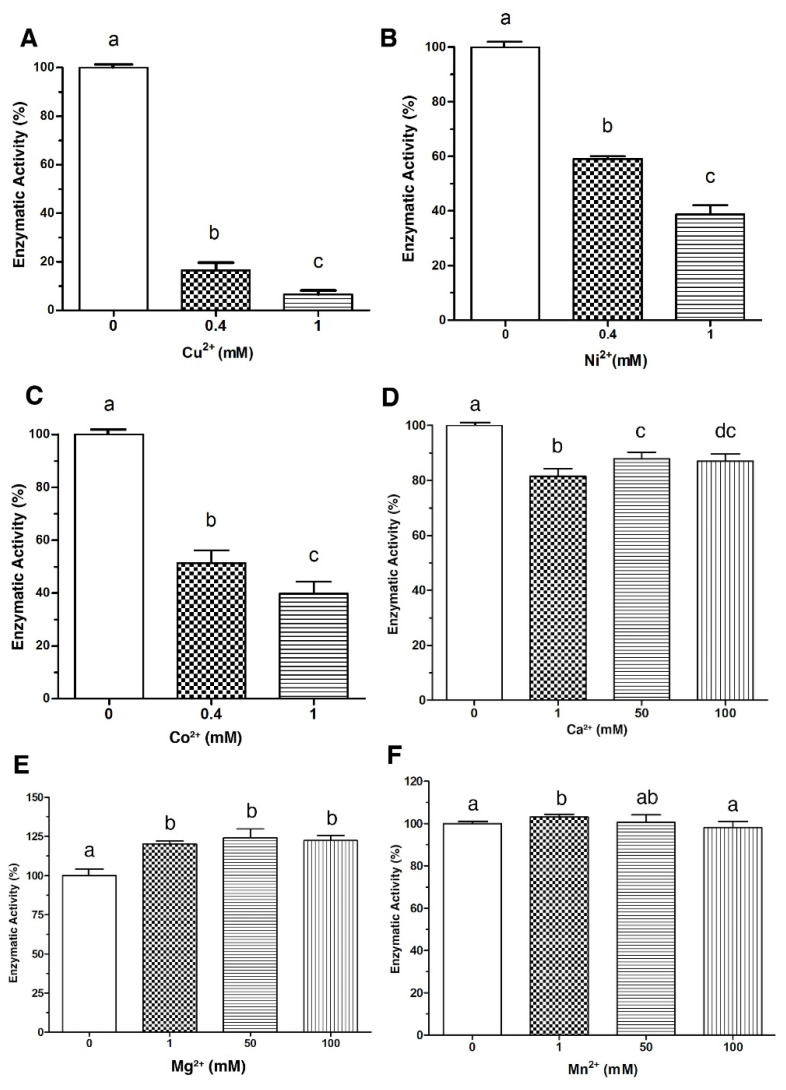
Effect of metal ions in the enzymatic activity of LDH. Enzymatic activity was assayed in the presence of divalent cations at the indicated concentration. Panel (**A**), Cu^2+^; Panel (**B**), Ni^2+^; Panel (**C**), Co^2+^, Panel (**D**), Ca^2+^; Panel (**E**), Mg^2+^ and Panel (**F**), Mn^2+^. Residual activity was calculated based on LDH activity under optimal assay conditions without metals. Results are mean ± SE (n = 3). Different letters indicate differences (*p* < 0.05) among treatments.

The enzymatic activity of LDH from *V. parahaemolyticus* in the presence of metal ions was investigated. As shown in Figure 1, Cu^2+^, Ni^2+^ and Co^2+^ inhibited (*p* < 0.05) the enzymatic activity of LDH in a dose-response manner. Cu^2+^ at 0.4 mmol/L inhibited the activity of LDH by 85%, while at 1 mmol/L reached up to 95% of inhibition (Figure 1A). Additionally, Ni^2+^ and Co^2+^ under the same conditions showed different effects; Ni^2+^ inhibited LDH by 40% and 60% of the total activity at 0.4 mmol/L and 1 mmol/L, respectively (Figure 1B), whereas Co^2+^ at the same concentrations inhibited 50% and 60% (Figure 1C). On the other hand, Ca^2+^ at 1 mmol/L reduced the activity only by 20%; nevertheless, increasing the concentration to 50 and 100 mmol/L caused ≈ 10% inhibition of the enzymatic activity (Figure 1D). While Mg^2+^ and Mn^2+^ increased LDH activity, Mg^2+^ (1 mmol/L) increased the LDH activity up to 26%, but higher concentrations (50 and 100 mmol/L) did not show an additional effect (*p* < 0.05) (Figure 1D). Additionally, Mn^2+^ slightly increased ≈ 5% the enzyme activity (*p* > 0.05). When the concentration of this ion was increased at 50 and 100 mmol/L, no differences (*p* > 0.05) were found compared to the control (without metal ions) (Figure 1F). 

**Figure 2:**
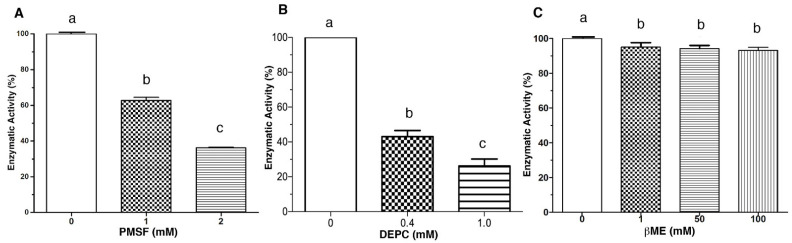
The effect of chemical agent in the enzymatic activity of LDH. Enzymatic activity was assayed in the presence of each compound at the indicated concentration. Panel (**A**), PMSF; Panel (**B**), DEPC and Panel (**C**), βME. Residual activity was calculated based on LDH activity under optimal assay conditions without metals. Results are mean ± SE (n = 3). Different letters indicate differences (*p* < 0.05) among treatments.

The effect of chemical modification reagents on LDH was also investigated. β-mercaptoethanol (βME), phenylmethylsulfonyl fluoride (PMSF), and diethyl pyrocarbonate (DEPC) were chosen due to their capacity to interact with functional groups of proteins. βME is used to reduce disulfide bonds, PMSF interacts preferentially with hydroxyl groups of serine residues, and DEPC can modify the imidazole group of histidine [21,22]. Figure 2 shows the effect of these compounds on the LDH enzymatic activity. PMSF significantly inhibited the LDH at the two evaluated concentrations (1 and 2 mmol/L) with 40% and 60% inhibition, respectively (Figure 2A). DEPC was the most effective compound to inhibit the enzyme, considering that 0.1 mmol/L caused a 60% decrement in activity. While increasing the concentration to 1 mmol/L, the enzymatic activity was reduced by 80% compared to the control (Figure 2B). In contrast, βME inhibits LDH only ≈10% at 1 mmol/L; when increasing the concentration of the reducing agent to 50 and 100 mmol/L, no inhibitory effect was observed (*p* < 0.05). Therefore, this compound was not a good enzyme inhibitor at the evaluated concentrations (Figure 2C).

### 2.2. Dose-Response Analysis of the Inhibition Effect Caused by Metal Ions and Chemical Reagents

**Figure 3:**
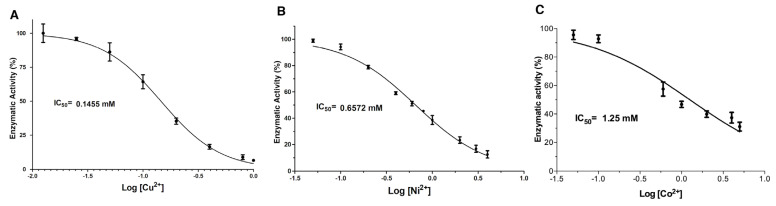
Dose-response analysis of LDH inhibition by metal ions. (**A**–**C**). Non-linear regression fitting of dose-response curves of LDH in the presence of each metal ion: Cu^2+^, Ni^2+^, and Co^2+^, respectively. Results are mean ± SE (n = 3).

A dose-response analysis was conducted to determine the concentration required to inhibit 50% enzyme activity (IC_50_). For this purpose, the ions Cu^2+^, Ni^2+^ and Co^2+^ and the chemical modification reagents DEPC and PMSF were selected based on the observed inhibitory activity (Figure 1 and Figure 2). The Cu^2+^ concentrations evaluated ranged from 0.0125 to 1 mmol/L; the enzymatic activity of LDH gradually decreased as the concentration of the inhibitor increased (Figure 3A). At low concentrations (<0.05 mmol/L), a slight decrease in the activity of 10% was observed. While at concentrations between 0.4 and 1 mmol/L, the maximum inhibition of enzymatic activity (>80%) was achieved. Non-linear regression fitting showed a IC_50_ = 0.1455 mmol/L (R^2^ = 0.95). Additionally, Ni^2+^ caused an inhibitory effect >50% at concentrations greater than 0.6 mmol/L; concentrations of 3 and 4 mmol/L reduced the activity of the LDH to less than 20% with IC_50_ = 0.6572 mmol/L (R^2^ = 0.98) (Figure 3B). Co^2+^ showed less capacity to inhibit LDH since the evaluated concentrations were greater than the other two ions mentioned above (0.05–5 mmol/L) (Figure 3E). Co^2+^ concentration was increased up to 5 mmol/L to achieve a 60% inhibition, which is 5 and 25 times higher than the concentration of Ni^2+^ and Cu^2+^ required to reach the same inhibitory effect, respectively. When increasing the concentration of Co^2+^ above 5 mmol/L, an LDH activity reduction was not observed. The data obtained were analyzed under the same conditions as the dose-response model, obtaining an IC_50_ value = 1.25 mmol/L (Figure 3C).

**Figure 4:**
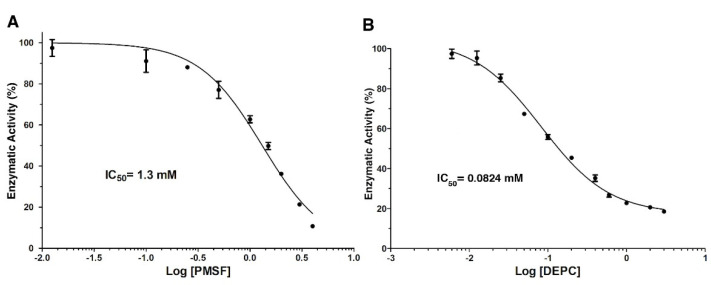
Dose-response analysis of LDH inhibition by chemical modification reagents. Panel (**A**) (PMSF) and (**B**) (DEPC) show the non-linear regression fitting of dose-response curves of LDH in the presence of each compound used to calculate IC_50_ values. Results are mean ± SE (n = 3).

On the other hand, Figure 4 shows the dose-response analysis for the chemical modification reagents, PMSF and DEPC. The LDH activity gradually decreased as PMSF concentration increased (Figure 4A). At 4 mmol/L of PMSF, the enzyme was inhibited by approximately 90%, in the absence of inhibitors. However, concentrations higher than 4 mmol/L caused the formation of a precipitate in the reaction cell. The data analysis in the dose-response model showed a correlation >0.95 with an IC_50_ value = 1.3 mmol/L (Figure 4A). 

Based on our previous results, DEPC was the most effective in inhibiting LDH activity; therefore, this chemical agent was evaluated in the range of 0.006–3 mmol/L (Figure 4B). This compound inhibited the enzyme by 40%, even at low concentrations (0.05 mmol/L) compared to the other ions and PMSF. The maximum inhibition (>80%) of LDH was observed at a concentration of 1 mmol/L. The activity of the enzyme remained practically constant when increasing (three times) the concentration of DEPC. Dose-response model fitting showed the lowest IC_50_ = 0.0824 mmol/L (R^2^ = 0.97) compared with other compounds (Figure 4B).

### 2.3. Effect of Metal Ions and Chemical Reagents on the LDH Hemolytic Activity

**Figure 5:**
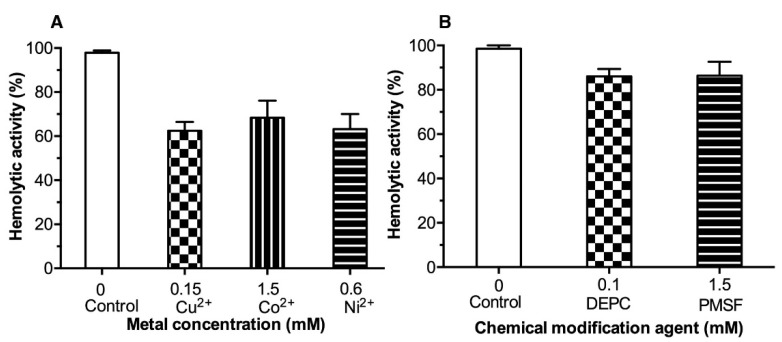
Inhibition of LDH hemolytic activity by metals (Panel (**A**) and chemical agents (Panel (**B**)). LDH hemolytic activity was calculated in the absence of the evaluated substances. Results are mean ± SE (n = 3).

Best inhibitors were chosen to inhibit hemolytic activity based on dose-response results in enzymatic assays. All three evaluated (Cu^2+^, Co^2+^, and Ni^2+^) metals inhibited the LDH hemolytic activity by similar values of 40, 30, and 37%, respectively. However, Cu^2+^ was the ion with the largest effect against LDH hemolytic activity (40% inhibition) with the lowest concentration (0.15 mmol/L) compared to Ni^2+^ (0.6 mmol/L) and Co^2+^ (1.5 mmol/L). Additionally, the inhibition of hemolytic activity by DEPC and PMSF were evaluated at 0.1 and 1.5 mmol/L, respectively (Figure 5A). DEPC (0.1 mmol/L) showed inhibition of ≈ 20%, while PMSF at a relatively higher concentration (1.5 mmol/L) achieved 15% inhibition compared to the control (Figure 5B).

## 3. Discussion

Recombinant LDH of *V. parahaemolyticus* was successfully refolded, showing esterase activity in the presence of lecithin as confirmed by the hydrolysis of PNPL described previously [15,24]. In the present work, the esterase activity of LDH was inhibited in the presence of metal cations; Cu^2+^ was the most effective inhibitor reducing its activity up to 95%, followed by Ni^2+^, Co^2+^, and Ca^2+^. Similarly, Jia et al. reported that Cu^2+^, Ni^2+^, and Co^2+^ at 0.1 mmol/L inhibited about 50% of the hemolytic activity of the LDH from *V. alginolyticus*, which shared 94% of its identity with the LDH of *V. parahaemolyticus* [11]. Both enzymes had phospholipase A_2_/lysophospholipase and hemolytic activity against human erythrocytes due to the release of lysophospholipids with high detergent capacity during the hydrolysis of glycerophospholipids at the sn-2 position [15,24]. On the other hand, previous studies highlighted the ability of divalent cations to inhibit different enzymes. Pérez-Legaspi and Rico-Martínez [25] demonstrated that Cu^2+^ inhibits the enzymatic action of a phospholipase A_2_ in vivo of three freshwater rotifers species. The authors mention that this metal ion was the most efficient inhibitor compared with cadmium, chromium, lead and titanium. Other enzymes are also susceptible to metal ions inhibition. Maheshwari and Dubey [26] reported a decrease in RNase activity from roots and shoots of rice seedlings when increasing Ni^2+^. The addition of 2500 μM NiSO_4_ or Ni(NO_3_)_2_ to the assay medium reduced about 49–59% and 33–84% RNase activity, respectively, concluding that the inhibitory of nickel could be related to interactions with functional groups of histidine in the active site [27]. 

**Figure 6:**
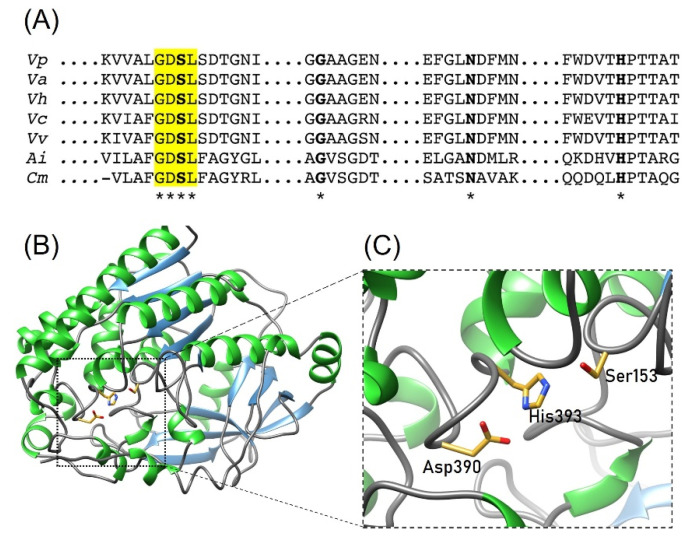
Conserved domain and catalytic residues of GDSL proteins. (**A**) Sequence alignment of GDSL hydrolases; the GDSL domain is highlighted in yellow and active site residues are in bold letters. * Indicates conserved residues in the active site among aligned sequences. (**B**) The overall structure of LDH and (**C**) Representation of the active site. The catalytic residues Ser153, Asp390 and His393 are indicated with sticks. The residue number is based on LDH from *V. parahaemolyticus.* Vp, *V. parahaemolyticus* (uniprot: BAA25328.1); Va, *V. alginolyticus* (C7EWQ8), Vh, *V. harveyi* (Q2XPT2); Vc, *V. cholera* (Q9KMV0); Vv, *V. vulnificus* (PDB ID: 6JL1); Ai, *Altererythrobacter indicus* (PDB ID: 7C82) and Cm, *Croceicoccus marinus* (PDB ID: 7C23).

The inhibitory effect of evaluated ions may be due to interactions with residues involved during the catalysis of LDH. It is well known that Cu^2+^, Ni^2+^, and Co^2+^ are attracted to histidine residues; thus, we hypothesize that these ions could interact with this residue (His393) located in the active site of LDH, causing a decrease in its activity. As mentioned before, LDH from *V. parahaemolyticus* has a GDSL motif characteristic of the hydrolase/esterase superfamily and well conserved SGNH hydrolases motif with a catalytic triad composed by Ser, His and Asn (Figure 6). In this sense, Cd^2+^ (10 mmol/L) reduced the enzymatic activity (80%) of AlinE4, an SGNH-hydrolase from *Altererythrobacter indicus* [28]. The crystal structure of AlinE4 showed that Cd^2^^+^ interacts with residues Ser13 and His165, which are located in the catalytic triad, suggesting that this divalent cation might act by blocking proton transfer and/or protecting substrates from nucleophile attack [28]. Therefore, we propose that a similar mechanism could occur in LDH from *V. parahaemolyticus* in the presence of ions evaluated in this work. However, structural studies are required to demonstrate this hypothesis. The inhibition degree difference of each ion could be due to the oxidation states of each one and the spatial conformation they take in the reaction [29,30,31]. 

On the other hand, an increase in the enzymatic activity of LDH in the presence of Mg^2+^ and Mn^2+^ was observed. These metal ions are commonly found as cofactors in other phospholipases [32]. Our results agree with other studies; Tingting et al. [33] reported that 1 mmol/L of Mn^2+^ activated Est19 from *Croceicoccus marinus*, an esterase member of the SGNH hydrolase family. Additionally, Garba et al. [34] reported that the presence of Mg^2+^ enhanced the enzymatic activity of PLA_2_ from *Naja nigricollis* venom. Mg^2+^ and Mn^2+^ stimulate the action of some phospholipases A in a dose-dependent way. This effect has been attributed to the substrate or enzyme physicochemical changes, such as the reduction of negative zeta potentials [35]. Additionally, metallic ions may interact with active site amino acids and stabilize the substrate during catalysis [34]. Thus, a similar effect could be responsible for increased LDH activity observed in the enzymatic assays. 

In the case of chemical modification reagents, LDH from *V. parahaemolyticus* seems more susceptible to inhibition of enzymatic and hemolytic activity by DEPC than the other evaluated compounds, since it shows a more drastic decrease in activity at relatively low concentrations (Figure 4B and Figure 5B). SGNH esterases/hydrolases contain a well-conserved catalytic residue (His393 in LDH from *V. parahaemolyticus*) located in block V that activates the residue Ser153 during catalysis [16,36]. DEPC can react with nitrogen atoms of the side chain of histidine residues [23]; thus, the inhibition observed with this compound may also be related to the interaction with the imidazole group of His393 in the active site. LDH has two important domains: GDSL-lipase/hydrolase and SGNH-hydrolase, where serine plays a catalytic role [36]. The SGNH domain is distributed among four conserved blocks, I, II, III and V, involved in the catalytic mechanisms of many esterases/hydrolases enzymes; Block I comprises the typical GXSXG motif found in lipases/esterases, in which Ser153 acts as the nucleophile during catalysis [16]. PMSF is frequently used as a protease inhibitor, which reacts with the hydroxyl group of serine residues [37]; the enzyme inhibition observed by this compound could be due to the modification of *V. parahaemolyticus* catalytic residue (Ser153). Our results showed that LDH enzymatic activity was inhibited by PMSF (60%) at 2 mmol/L, (IC_50_ = 1.3 mmol/L), while hemolytic activity was reduced by 10% at a similar concentration to IC_50_ value (1.5 mmol/L). Thus, evidencing the importance of the serine residue (Ser153) in *V. parahaemolyticus* LDH. Additionally, Jia et al. [11] reported that DEPC inhibited the hemolytic activity of LDH from *Vibrio alginolyticus* by 50% at 0.1 and the increase of the concentration to 1 mmol/L and 10 mmol/L did not cause additional inhibition, whereas, for PMSF, 50% of inhibition was observed at 0.1 mmol/L; nevertheless, higher concentrations showed less inhibitory effect with values of 25% and 12.5% at 1 and 10 mmol/L, respectively [11]. Conversely, Chakraborti and Michael [38] used PMSF to investigate biochemical mechanisms associated with the oxidant-caused activation of serine esterase PLA_2_ in bovine lung cells and reported that the activity of this enzyme was reduced by about 30% using a PMSF at 1 mmol/L. Regarding βME, a low inhibitory effect was observed (≈10%) at the evaluated concentrations (1–100 mmol/L). The crystal structure of LDH from *V. vulnificus* (PDB ID: 6JL1) exhibits two disulfide bridges involving Cys48-Cys103 and Cys356-Cys377 [34]. Sequence alignment shows that Cys residues are conserved in LDH from *V. parahaemolyticus*; thus, the lack of inhibitory effect of βME suggests that no S-S bonds are formed in this enzyme. However, structural and biophysical studies are needed to provide more evidence about the role of Cys residues in LDH function and stability.

On the other hand, the resulting IC_50_ agrees with IC_50_ values reported for enzymes of the PLA_2_ family and other inhibitors at a concentration range from 0.01 to 0.5 mmol/L [39,40,41,42]. It is important to mention that up to our literature revision, we have not found another study where the IC_50_ values for the LDH of other *Vibrio* species are reported. Based on these results, both DEPC and PMSF could inhibit enzymatic and hemolytic activity of LDH from *V. parahaemolyticus*, which could be by interaction with amino acid residues related to the active site as Ser153 and His393. Future investigation should focus on demonstrating the role of chemical agents in the modification or interaction with active site residues of LDH from *V. parahaemolyticus*.

## 4. Conclusions

Our results showed that the enzymatic activity of recombinant LDH from *V. parahaemolyticus* was inhibited by metal ions Cu^2+^, Co^2+^ and Ni^2+^ and by chemical modification using DEPC and PMSF. Molecular modeling suggests that the SGNH domain shows a conserved spatial distribution as reported in other GDSL hydrolases. Therefore, metal ions and chemical agents could interact or modify important catalytic amino acid residues, such as Ser153 and His393. Both biophysics and structural studies could contribute to understanding the mechanism of inhibition of LDH from *V. parahaemolyticus*.

## 5. Materials and Methods

### 5.1. LDH Protein Expression and Purification

Recombinant LDH from *V. parahaemolyticus* was obtained as previously described by Vazquez-Morado et al. [15]. From the nucleotide sequence encodes LDH (Accession number BAA25328.1), a synthetic gene with C-terminal 6x-His tag cloned into pET-28b (+) plasmid. Chemically competent *Escherichia coli* Rosetta 2 strains were transformed with this vector by heat shock and incubated in SOC media (tryptone 2% *w*/*v*, yeast extract 0.5% *w*/*v*, 10 mmol/L NaCl, 2.5 mmol/L KCl, 10 mmol/L MgCl_2_, 10 mmol/L MgSO_4_ and 20 mmol/L glucose) at 37 °C by 4 h. For plasmid selection, bacterial cells were plated on Luria-Bertani agar plates supplemented with kanamycin (25 µg/mL) at 37 °C overnight.

The transformed strains were cultivated in LB broth supplemented with kanamycin (50 µg/mL) at 37 °C with constant agitation at 220 rpm in a MaxQ 4000 orbital shaker (Thermo-Scientific, Waltham, MA, USA). When the optical density reached O.D. = 0.6 (λ = 600 nm), IPTG (Isopropyl β-D-1-thiogalactopyranoside) was added to a final concentration of 1 mmol/L to induce the overexpression of LDH (16 h at 25 °C, 220 rpm). Subsequently, the biomass was recovered by centrifugation (7000 rpm for 20 min at 4 °C), pelleted and stored at −80 °C until use.

To purify LDH, 1 g of biomass was resuspended in lysis buffer (50 mmol/L Tris base, 5 mmol/L EDTA, 100 mmol/L NaCl, 6 mmol/L benzamidine and 1 mmol/L DTT) in a 1:4 ratio. Bacterial cells were lysed by sonication on an ice bed with six pulses of 10-s and 40-s rest at 30% amplitude. The homogenate was clarified at 12,000 rpm for 20 min at 4 °C, and SDS-PAGE (12%) stained with blue-coomassie was used to analyze the protein expression in soluble and insoluble fractions. 

Inclusion bodies were isolated from insoluble cellular debris, which was resuspended by sonication (as before) using buffer 1 (50 mmol/L Tris base, 1 mmol/L DTT, 5 mmol/L EDTA, 2% Triton X-100; pH 7.0) at a ratio of 1:8 (*w*/*v*), and the homogenate was centrifugated at 12,000 rpm for 20 min. The precipitate was recovered, and centrifugation was repeated three times. After that, the pellet was washed two times in buffer 2 (buffer 1, without Triton X-100) under the same conditions. Finally, the recovered inclusion bodies were solubilized using urea (50 mmol/L Tris base, 1 mmol/L DTT, 8 M urea, pH 7.0) by sonication and incubated overnight at 4 °C with constant stirring for 12 h. The homogenate was clarified (12,000 rpm at 4 °C for 30 min), and the soluble fraction containing the LDH in 8 M urea was recovered. The protein concentration was quantified at 280 nm in nanodrop equipment considering the molar extinction coefficient (ϵ) ≈ 96,510 M^−1^ cm^−1^.

Finally, the LDH was purified by Immobilized Metal Affinity Chromatography (IMAC) under denaturing conditions (8 M urea) and on a 1 mL HiTrap™ affinity column using the Äkta Prime Plus equipment (GE Healthcare, Chicago, IL, USA). LDH was refolded by dialysis on a 12 kDa membrane until urea was removed. The purity and concentration of the enzyme were determined by SDS-PAGE and the Bradford method, respectively.

### 5.2. Enzymatic Activity and Hemolytic Assay

Enzymatic activity was measured spectrophotometrically using the lipase/esterase assay described previously by Vazquez-Morado et al. [15], using p-nitrophenyl laurate (PNPL) as substrate. Each reaction was conducted in a final volume of 1 mL containing: 50 mmol/L Tris-HCl pH 8.0, 0.2 mmol/L PNPL and refolded LDH (6.2 µM final concentration). The reaction was started by adding egg yolk lecithin (0.0001% final concentration).

PNPL hydrolysis was monitored by reading the absorbance at 410 nm every 5 s for 2 min. The reaction was carried out in a quartz cell in a Cary 50^®^UV-VIS spectrophotometer (Varian). The negative control assay consisted of the same reaction without LDH. One unit of lipase activity is defined as the amount of enzyme required to release 1 µmol of p-nitrophenol per minute. Specific activity was calculated using the equation:

U/(mg of protein) = (m⋅V)/(ε⋅p⋅l)(1)

Where m is the slope of the reaction, V is the reaction volume, p is the protein concentration (mg/mL) and l is the cell path length in cm.

The hemolytic activity of LDH was quantified using human erythrocytes as a substrate, according to Vazquez-Morado (2021) [15]. Erythrocytes were extracted from human blood, donated by one volunteer, previously informed about the procedure; the sample was used, and properly discareded following the established protocols by the Institutional program of environmental health and safety of the Universidad de Sonora. Additionally, the volunteer provided signed informed consent about participating in this research. 

Erythrocytes were obtained from human blood extracted by peripheral venipuncture using 6 mL BD Vacutainer^®^ tubes with a coagulation activator and then centrifuged (700 rpm for 5 min at 4 °C). The erythrocytes were washed (three times) with sterile saline solution (0.9%), and centrifuged for 5 min at 4000 rpm, discarding the supernatant. After this, the erythrocytes were resuspended in PBS buffer in a 1:20 ratio. The hemolytic assay was performed in microtubes, where 1× PBS buffer, erythrocyte suspension, LDH (0.23 mM final concentration) and egg yolk lecithin 0.0006% were added, carefully homogenized and incubated at 37 °C for 30 min. After that, samples were centrifuged for 1 min at 6500 rpm and the supernatant was transferred to a microplate to measure the absorbance at 405 nm using the Multiskan GO equipment (Thermo Fisher Scientific, Tokyo, Japan). The lysis of the erythrocytes by tween 20 was taken as a positive control (100% hemolysis). All the tests were performed in triplicate and expressed as a percentage of hemolytic activity.

### 5.3. Inhibition of Enzymatic and Hemolytic Activity by Metal Ions and Chemical Modification Reagents

To evaluate the effect of metal ions on LDH enzymatic activity, Cu^2+^ (0.0125 to 1 mmol/L), Ni^2+^ (0.05 to 4 mmol/L), Co^2+^ (0.05 to 5 mmol/L), Mn^2+^, Ca^2+^ and Mn^2+^ (1, 50 and 100 mmol/L) were used. To determine functional groups involved in the LDH enzymatic activity, the chemical modification reagents used were diethyl pyrocarbonate (DEPC; 0.006 to 3 mmol/L), phenylmethylsulfonyl fluoride (PMSF; 0.0125 to 4 mmol/L) and β-mercaptoethanol (βME; 1, 50 and 100 mmol/L). As described above, metal ions and chemical modification reagents at different concentrations were added to the quartz cell before starting the reaction. All assays were performed in triplicate. LDH activity without metal ions or chemical reagents was a positive control.

The inhibitory effect of each metallic ion on LDH hemolytic activity was performed using the same procedure described before and adding Cu^2+^, Co^2+^ and Ni^2+^ at 0.15, 1.5 and 0.6 mmol/L, respectively. These concentrations were based on results obtained during enzymatic inhibition assays (IC_50_ values). Negative control was the reaction solution without enzyme but with metal ions to determine the true effect created by LDH and not by the different ions. Positive control was the reaction solution with the enzyme without metals. 

On the other hand, to investigate the participation of chemical modifying agents on LDH hemolytic activity, IC_50_ values obtained in dose-response analysis for best inhibitors were chosen: DEPC (0.1 mmol/L) and PMSF (1.5 mmol/L). In the same way, all assays were carried out under the protocols mentioned in the hemolytic activity section and expressed as percentage of hemolytic activity; the reaction solution without chemical modification reagents (100% hemolytic activity) was used as positive control. All measurements were performed in triplicate.

### 5.4. Dose-Response Analysis

To determine the concentration required to inhibit 50% of enzyme activity (IC_50_), the ions and chemical reagents with the most inhibitory activity against LDH were selected. IC_50_ determination was carried out by varying the concentration of the chosen treatments in an interval that allowed fitting the data to a dose-response model by non-linear regression. This analysis was performed using the Prism 5 software (Graphpad^®^).

### 5.5. Structural Modeling

Model building was developed using the Phyre2 server in intensive mode with the one-to-one modeling function [43] using LDH from *V. vulnificus* (PDB ID: 6JL1) as a template, which has a sequence identity of >74% to LDH from *V. parahaemolyticus.* The obtained model was visualized with the UCSF Chimera software [44].

### 5.6. Statistical Analysis

Statistical analysis was performed to evaluate and compare the different concentrations used in the inhibition assays. The Mann–Whitney U test was applied by using the IBM SPSS Statistics 23 program. Significant differences were considered with a value of *p* < 0.05.

## Data Availability

Not applicable.
